# Familial relative risks for breast cancer by pathological subtype: a population-based cohort study

**DOI:** 10.1186/bcr2476

**Published:** 2010-02-10

**Authors:** Nasim Mavaddat, Paul D Pharoah, Fiona Blows, Kristy E Driver, Elena Provenzano, Deborah Thompson, Robert J MacInnis, Mitul Shah, The SEARCH Team, Douglas F Easton, Antonis C Antoniou

**Affiliations:** 1 Cancer Research UK, Genetic Epidemiology Unit, Department of Public Health and Primary Care, University of Cambridge, Strangeways Research Laboratory, Worts Causeway, Cambridge, CB1 8RN, UK; 2 Department of Oncology, University of Cambridge, Strangeways Research Laboratory, Worts Causeway, Cambridge, CB1 8RN, UK; 3 Addenbrookes Hospital NHS Trust, Hills Rd, Cambridge, CB2 0QQ, UK; 4 Centre for Molecular, Environmental, Genetic and Analytic Epidemiology, The University of Melbourne, 723 Swanston Street, Carlton, VIC 3053, Australia

## Abstract

**Introduction:**

The risk of breast cancer to first degree relatives of breast cancer patients is approximately twice that of the general population. Breast cancer, however, is a heterogeneous disease and it is plausible that the familial relative risk (FRR) for breast cancer may differ by the pathological subtype of the tumour. The contribution of genetic variants associated with breast cancer susceptibility to the subtype-specific FRR is still unclear.

**Methods:**

We computed breast cancer FRR for subtypes of breast cancer by comparing breast cancer incidence in relatives of breast cancer cases from a population-based series with known estrogen receptor (ER), progesterone receptor (PR) or human epidermal growth factor receptor 2 (HER2) status with that expected from the general population. We estimated the contribution to the FRR of genetic variants associated with breast cancer susceptibility using subtype-specific genotypic relative risks and allele frequencies for each variant.

**Results:**

At least one marker was measured for 4,590 breast cancer cases, who reported 9,014 affected and unaffected first-degree female relatives. There was no difference between the breast cancer FRR for relatives of patients with ER-negative (FRR = 1.78, 95% confidence intervals (CI): 1.44 to 2.11) and ER-positive disease (1.82, 95% CI: 1.67 to 1.98), P = 0.99. There was some suggestion that the breast cancer FRR for relatives of patients with ER-negative disease was higher than that for ER-positive disease for ages of the relative less than 50 years old (FRR = 2.96, 95% CI: 2.04 to 3.87; and 2.05, 95% CI: 1.70 to 2.40 respectively; P = 0.07), and that the breast cancer FRR for relatives of patients with ER-positive disease was higher than for ER-negative disease when the age of the relative was greater than 50 years (FRR = 1.76, 95% CI: 1.59 to 1.93; and 1.41, 95% CI: 1.08 to 1.74 respectively, P = 0.06). We estimated that mutations in BRCA1 and BRCA2 explain 32% of breast cancer FRR for relatives of patients with ER-negative and 9.4% of the breast cancer FRR for relatives of patients with ER-positive disease. Twelve recently identified common breast cancer susceptibility variants were estimated to explain 1.9% and 9.6% of the FRR to relatives of patients with ER-negative and ER-positive disease respectively.

**Conclusions:**

FRR for breast cancer was significantly increased for both ER-negative and ER-positive disease. Including receptor status in conjunction with genetic status may aid risk prediction in women with a family history.

## Introduction

Family history is a well established risk factor for breast cancer, with the familial relative risk (FRR) being approximately two-fold for first degree relatives of breast cancer patients compared with controls from the general population [1]. FRR for breast cancer varies both with age of cancer diagnosis of the index case and the age of the relative, and increases with the number of affected relatives [1,2]. The risk is higher in monozygotic twins of breast cancer cases than dizygotic twins [3]. Moreover, none of the known environmental risk factors for breast cancer appear to influence FRR [1]. These findings suggest that FRR for breast cancer is a direct reflection of the genetic component of the disease [3,4]. High risk alleles such as *BRCA1 *and *BRCA2 *explain less than 20% of the FRR [4] and the residual familial risk is best described by a polygenic model comprising multiple variants, each of modest risk [5]. This model is supported by the recent discovery of common low risk variants through genome wide association studies (GWAS) [6-12]. Interestingly, most of the genetic variants associated with breast cancer susceptibility discovered to date have stronger associations with estrogen receptor (ER)-positive than ER-negative breast cancer [9,10,13,14]. Conversely tumours in *BRCA1 *mutation-carriers are more likely to be ER-negative than tumours in non-carriers [15]. It is plausible therefore that FRR of breast cancer varies by pathological characteristics of the tumour of the index case.

The division of breast cancer into ER-negative and ER-positive disease is well-established. These subtypes have been differentiated for example, by their distinct age-specific incidence patterns and prognosis [16]. Several major sub-types of breast cancer can also be defined on the basis of joint expression of three immunohistochemical markers commonly used in clinical practice - ER, PR and HER2. Luminal tumours are those that express either ER or PR. These can be divided according to HER2 expression into HER2 expressing and non-expressing phenotypes. The non-luminal tumours are ER- and PR- negative and may be divided into HER2 expressing and triple negative (TN) tumours. The TN phenotype is often regarded as synonymous with the basal type tumours as classified by gene expression studies, but several studies have shown that the TN tumours that express basal markers are different from those that do not [17,18]. Basal-like breast cancer defined by five biomarkers has superior prognostic value than triple-negative phenotype [19]. Basal type tumours are particularly prevalent in *BRCA1 *associated breast cancer [20].

We used a large population based case series, Studies of Epidemiology and Risk factors in Cancer Heredity (SEARCH), and a retrospective cohort design to compute FRRs separately for relatives of patients with different tumour subtypes, primarily ER-positive and ER-negative disease, but also subtypes defined by PR and HER2, where data were available. Subtype information was available only for the tumour diagnosed in the proband, but not for tumours diagnosed in the relatives. Breast cancer FRRs for each subtype, were calculated by comparing the incidence of breast cancer in relatives of cases with published incidences in the general population. We further estimated the contribution of the breast cancer susceptibility genes identified to date to the subtype specific FRR. Knowledge of the FRR for breast cancer by disease subtype can provide insights into genetic predisposition to ER, PR and HER2 specific disease. Such information can also be incorporated in risk algorithms aimed at estimating the risk of developing breast cancer [21,22].

## Materials and methods

### Study participants

Study participants were selected as described by Pharoah et al. [23]. In brief, data on breast cancer patients were drawn from Studies of Epidemiology and Risk factors in Cancer Heredity (SEARCH), an ongoing population-based study with cases ascertained through the Eastern Cancer Registration and Information Centre (ECRIC). All patients diagnosed with invasive breast cancer below age 55 years since 1991 and still alive in 1996 (prevalent cases, median age 48 years), together with all those diagnosed below age 70 years between 1996 and the present (incident cases, median age 54 years) were eligible to take part. The ethnic background, as reported on the questionnaires, was white for 98% of cases. Study participants were invited to complete an epidemiological questionnaire that included questions on family history, reproductive history, oral contraceptive and hormone replacement therapy use, past medical history and previous examination of the breast by mammography. The family history data included details of all first degree relatives and grandparents, their date and place of birth, date of death and any history of cancer. Details of any other relatives known by the case to have had cancer were also ascertained. Ascertainment of cancers in relatives was not independently confirmed through reference to cancer registry data.

Tissue micro-arrays were constructed from tumours for 2,659 of the cases. These were stained for ER, PR and HER2 and scored as shown in Table S1 in Additional file 1. Additional data on ER status was obtained through ECRIC from routine pathology and clinical records. Tissue micro-array data were preferentially used when ER status as determined by TMA and obtained from the medical records were discordant (Table S2 in Additional file 1). The study was approved by the Eastern Region Multi-centre Research Ethics Committee, and all patients gave written informed consent. *BRCA1 *and *BRCA2 *mutation screening had been previously carried out in the entire prevalent series of patients and details are provided elsewhere [4].

### Statistical methods

Breast cancer FRRs were estimated from a cohort analysis of the first degree relatives of SEARCH index cases, as described by Pharoah et al [23]. At risk women entered the cohort on 1 January 1960 and were censored on the first of the following events: any cancer diagnosis, death, the date the family history questionnaire was completed or age 85. Relatives whose dates of birth were unknown (6% of relatives) were given a date based on their relationship to the index case. For example, parents were given a date of birth 30 years before the date of birth of the index case, and sisters were given the same date of birth as the index case. Relatives born before 1890 were excluded from the analysis. As no differences in the age specific FRR between prevalent (20% of cases) and incident cases was found in the study of Pharoah et al [23], prevalent and incident cases were combined in this study. For our purposes, first degree relatives include only sisters and mothers as very few cases in daughters were recorded.

The expected numbers of cancers among the first degree relatives were computed from national age- sex- and period-specific incidence rates for England and Wales, as published in Cancer in Five Continents [24-30]. 1 January 1960 was used as a cut-off for entry into the cohort because national incidence rates prior to this date are unreliable. FRRs were computed separately for each pathological subtype from the ratio of observed number of breast cancers to the expected number of breast cancers. Since subtype information was only available for the index case but not for tumours in their relatives, these FRRs therefore represent the probability of developing breast cancer of any subtype given the relative developed breast cancer of a particular subtype, compared with the probability of developing breast cancer (of any type) in the general population. Subtypes were defined as luminal (ER and/or PR positive), luminal HER2-positive, luminal HER2-negative, non-luminal HER2-positive (ER and PR-negative, HER2-positive), and TN (ER, PR and HER2-negative). Data were also analysed by individual marker status and joint expression of ER and PR. Where sufficient data were available, analyses were further subdivided by the age at diagnosis of the index case, or by following up relatives either 1) from birth up until censoring or age 50, whichever occurred first or 2) from age 50 to censoring. To address the dependency between individuals belonging to the same family, robust standard errors were used to calculate 95% confidence intervals (CI) and for tests of statistical significance [31]. All statistical analyses were carried out in Stata v.10.

### Contribution of recently identified susceptibility loci to FRR

The relative risk to daughters of an affected individual attributable to a given single nucleotide polymorphism (SNP) (ν*) was calculated using the formula [32]:

Where *p *is the population frequency of the minor allele, *q *= 1 - *p*, and *r*_1 _and *r*_2 _are the subtype-specific genotypic relative risks (estimated as odds ratio (OR)) for heterozygotes and rare homozygotes, relative to common homozygotes. For these calculations concordance in tumour type between proband and their relatives is assumed.

The proportion of the familial risk attributable to the SNP was then calculated as  where λ_0 _is the FRR for breast cancer (of any type) to first degree relatives of cancer cases with each subtype, as estimated from our study. This formula assumes that the SNP of interest and other susceptibility alleles act multiplicatively on risk. Estimates of *r*_1 _and *r*_2 _and minor allele frequency for populations of European ancestry for each susceptibility allele were derived from the literature. Only SNPs that had been genotyped in breast cancer cases from Caucasian populations were included in the analysis.

### Contribution of BRCA1 and BRCA2 to the FRR

We have recently extended the Breast and Ovarian Analysis of Disease Incidence and Carrier Estimation Algorithm (BOADICEA) breast cancer risk prediction software to incorporate tumour pathology information ([22] and Mavaddat et al. in preparation, 2009). The contribution of *BRCA1 *and *BRCA2 *mutations to the FRR for breast cancer was estimated as above by modelling the FRR due to each of the genes using the extended BOADICEA algorithm [21,22]. Briefly, the age specific FRR (FRR(t)) of breast cancer (BC) to daughters of an individual diagnosed with ER-negative disease was calculated as the ratio of pedigree likelihoods obtained from the extended BOADICEA, which is implemented in the pedigree analysis software MENDEL [21,22]:

To calculate an overall FRR, the age-specific FRRs were averaged over ages 20 to 70 years, weighted by the age distribution of breast cancer cases in the population.

To obtain the FRR attributable to *BRCA1 *or *BRCA2 *mutations separately, we assumed a reduced genetic model where only the relevant mutation conferred increased breast cancer risks and no other genetic effects were assumed in the model. We also estimated the subtype specific FRR, assuming that concordance in tumour pathology between the index case and relative was only due to genotype.

## Results

### Reported family history of breast cancer and pathology of index case tumours

Data from 7,338 breast cancer cases recruited into the SEARCH study were available for this analysis. These women reported a total of 14,439 affected and unaffected sisters and mothers. A history of breast cancer in any first degree female relative was reported in 1,163 (15.9%) cases. Seventy-four women (approximately 1%) reported two or more affected first degree relatives. ER status was available in 4,529 (62%), PR status in 2,478 (34%) and HER2 status in 1,896 (26%) of breast tumours from the index case. After adjusting for age, there was no significant difference in expression of tumour markers between prevalent and incident breast cancer cases (data not shown, all *P *> 0.05). Pathological information was missing in a slightly higher proportion of tumours from younger patients. There was no relationship between missing marker status and reporting of family history (data not shown, all *P *> 0.05). Table 1 shows the numbers and percentages of index case tumours (among all those tested) in each phenotypic group by family history (that is, a reported diagnosis of breast cancer in a first degree relative). The proportions of cases with and without family history were similar for each of the pathological subtypes analysed. Logistic regression analysis, treating receptor status (positive/negative) as the outcome variable and family history as the explanatory variable and adjusting for age at diagnosis revealed no associations between family history and the receptor status of the index case (Table 1).

**Table 1:** Index case tumour subtypes and distribution of cases according to reported family history

Phenotype of index case tumour	Number of tumours (%)**	**Number with affected relative (%)**^**†**^	**Number without affected relative (%)**^**‡**^	Comparison group for case-only analysis	**OR**^**§**^	95%CI	*P*-valuee
ER-positive	3659 (81)	599 (82)	3060 (81)	ER-negative	1.04	0.85-1.28	0.70
PR-positive	1709 (69)	267 (69)	1442 (69)	PR-negative	1.01	0.79-1.27	0.96
HER2-positive	228 (12)	38 (13)	190 (12)	HER2-negative	1.15	0.79-1.68	0.78
ER+PR+	1581 (65)	247 (64)	1334 (65)	ER-PR-	1.00	0.75-1.31	0.95
ER-PR+	110 (4.5)	18 (5)	92 (5)				
ER+PR-	261 (11)	43 (11)	218 (11)				
ER or PR+ (luminal)	3787 (83)	619 (84)	3168 (83)				
ER or PR+ HER2+ (luminal HER2+)	138 (7)	23 (8)	115 (7)	luminal HER2-	1.17	0.73-1.88	0.51
ER or PR+ HER2- (luminal HER2-)	1404 (75)	209 (74)	1195 (75)				
ER-PR-HER2+ (non-luminal HER2+)	76 (5)	11 (4)	65 (5)	TN	1.02	0.48-2.16	0.96
ER-PR-HER2- (TN)	218 (13)	30 (12)	188 (13)				

ER: estrogen receptor; HER2: human epidermal growth factor receptor; PR: progesterone receptor; TN: triple negative.

* reported diagnosis of breast cancer in a first degree relative

** Number of tumours in phenotypic group as a percentage of those tested for that phenotype

† Number of tumours in phenotypic group with an affected first degree relative as a percentage of cancers with measured phenotype and an affected first degree relative

‡ Number of tumours in phenotypic group without any affected first degree relative as a percentage of cancers with measured phenotype and without any affected relative

^§^OR, 95% CI (confidence intervals) and *P*-values associated with family history of breast cancer in any first degree relative, adjusting for age at diagnosis of the index case.

### Familial relative risks by pathological subtype

Table 2 summarises the estimated familial relative risks by pathological subtype. The relative risk of breast cancer for first degree relatives of a breast cancer case was estimated to be 1.78 (95% CI: 1.68 to 1.89). The estimated FRR for breast cancer for relatives of patients with ER-negative disease was 1.78 (95% CI: 1.44 to 2.11) and for relatives of patients with ER-positive disease 1.82 (95% CI: 1.67 to 1.98). For sisters of index cases the FRR was 2.01 (95% CI: 1.81 to 2.21) and for mothers 1.66 (95% CI: 1.53 to 1.79). The risk of breast cancer in sisters of ER-positive cases was greater than the corresponding risk in mothers (2.12 vs 1.67) but the statistical evidence for heterogeneity was weak (*P *= 0.08). There was little difference in the breast cancer FRR for mothers and sisters of ER-negative disease patients (*P *= 0.23).

**Table 2:** Familial relative risk for breast cancer by ER status of the index case tumour

	OBS	EXP	FRR	95%CI
Mothers				
All cases	663	398.81	1.66	1.53 - 1.79
ER-negative	84	45.09	1.86	1.46 - 2.27
ER-positive	332	199.36	1.67	1.49 - 1.85
Sisters				
All cases	416	207.25	2.01	1.81 - 2.21
ER-negative	37	22.97	1.61	1.05 - 2.17
ER-positive	226	106.81	2.12	1.83 - 2.41
All relatives				
All cases	1079	606.06	1.78	1.68 - 1.89
ER-negative	121	68.06	1.78	1.44 - 2.11
ER-positive	558	306.17	1.82	1.67 - 1.98

CI: confidence interval; ER: estrogen receptor; EXP: expected, FRR: familial relative risk; OBS: observed.

**Figure 1:**
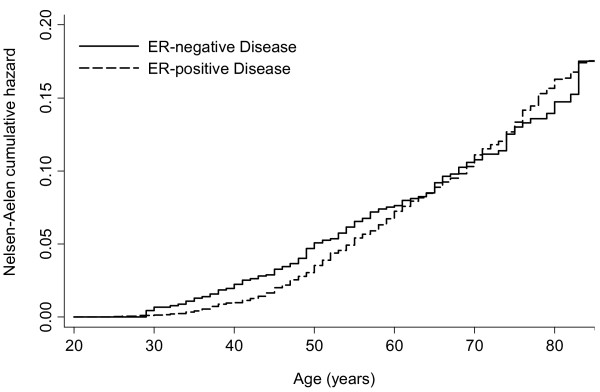
**Nelson-Aalen cumulative hazard estimate for breast cancer in relatives of ER-positive and ER-negative cases**.

The effect of age at diagnosis of the index patient and attained age of the relative on FRR for age categories less than 50 and 50 years and older is shown in Table 3. The overall FRR declines as both the age at diagnosis of the index patient and the age of the relative increase, and this effect is generally observed in both ER-positive and ER-negative groups (Table 3). The highest FRR was observed in relatives of ER-negative patients where both age of diagnosis of relative and age at diagnosis of index case was less than 50 years old (FRR 3.40 (95% CI: 2.04 to 4.75)). The FRR was even higher when the age at diagnosis of the index case was less than 35 years (FRR 8.26 (95% CI: 3.93 to 17.32)). The breast cancer FRR for relatives of ER-negative cases was higher than the FRR for relatives of ER-positive cases for ages of the relative less than 50 years old (FRR 2.96 (95% CI: 2.04 to 3.87) and 2.05 (95% CI: 1.70 to 2.40) respectively) but the difference was not significant (*P *= 0.07). The FRR for relatives of ER-positive cases was somewhat higher than the FRR for relatives of ER-negative cases when the age of the relative was greater than 50 years (FRR 1.76 (95% CI: 1.59 to 1.93) and 1.41 (95% CI 1.08 to 1.74) respectively) but again the difference was not significant (*P *= 0.06). These trends are also illustrated in Figure 1, which shows Nelson-Aalen cumulative hazard curves for breast cancer in relatives of cases with ER-negative and ER-positive disease.

**Table 3:** Age-specific familial relative risk for breast cancer by ER status of the index case tumour

Age of relative (years) †	ER-negative				ER-positive				All cases			
	
	OBS	EXP	FRR	95%CI	OBS	EXP	FRR	95%CI	OBS	EXP	FRR	95%CI
Index case <50*												
<50†	26	7.65	3.40	2.04-4.75	60	22.21	2.70	2.01-3.39	129	50.62	2.55	2.09-3.01
50 to 84	21	14.77	1.42	0.81-2.03	84	50.3	1.67	1.32-2.02	186	113.76	1.64	1.33-1.95
0 to 84	47	22.43	2.10	1.48-2.71	144	72.51	1.99	1.65-2.32	315	164.37	1.92	1.70-2.13
**Index case >= 50**												
<50	22	8.59	2.56	1.31-3.81	75	43.57	1.72	1.33-2.11	160	82.16	1.95	1.63-2.56
50 to 84	52	37.04	1.40	1.01-1.80	339	190.09	1.78	1.59-1.98	604	359.53	1.68	1.54-1.82
0 to 84	74	45.63	1.62	1.22-2.02	414	233.66	1.77	1.59-1.95	764	441.69	1.73	1.60-1.86
**Index case any age**												
<50	48	16.24	2.96	2.04-3.87	135	65.78	2.05	1.70-2.40	289	132.78	2.18	1.92-2.44
50 to 84	73	51.82	1.41	1.08-1.74	423	240.39	1.76	1.59-1.93	790	473.28	1.67	1.54-1.80
0 to 84	121	68.06	1.78	1.44-2.11	558	306.17	1.82	1.67-1.98	1079	606.06	1.78	1.68-1.89

CI: Confidence Interval; ER; estrogen receptor; EXP: expected; FRR: familial relative risk; OBS: observed.

* age at which index is affected

† age of relative (years) <50: relatives were followed up from birth up until censoring or age 50, whichever occurred first, 50 to 84: from age 50 to censoring, 0 to 85: from birth to censoring

The FRR for breast cancer by other pathological subtypes of tumours in index patients are shown in Table 4. Due to limited data, the analyses were not subdivided by the age of the index case. The FRR for relatives of PR-negative disease was higher than the FRR for relatives of PR-positive disease for ages of the relative less than 50 years old (*P *= 0.02). The breast cancer FRR for relatives of HER2-positive cases was higher than for relatives of HER2-negative cases regardless of the age of the relative (2.02 (95% CI: 1.34 to 2.69) and 1.69 (95% CI: 1.46 to 1.92) respectively), but there was little statistical evidence for heterogeneity (*P *= 0.5). There were increased FRRs for breast cancer associated with all subtypes defined by joint ER, PR and HER2 status. There was no significantly increased FRR for breast cancer in relatives of patients with TN disease, but the number of patients with luminal HER2+ (n = 138), non-luminal HER2+ (n = 76) and TN tumours (n = 218) were too small to draw definitive conclusions.

**Table 4:** Familial relative risk for breast cancer by marker status of index case tumour and age of relative *

Phenotype	Age of relative (years)*	OBS	EXP	FRR	95%CI
**PR-positive**					
	<50	60	29.46	2.04	1.52 to 2.55
	50 to 84	187	102.77	1.82	1.55 to 2.10
	0 to 84	247	132.23	1.87	1.63 to 2.11
**PR-negative**					
	<50	46	13.39	3.44	2.37 to 4.50
	50 to 84	65	46.00	1.41	1.06 to 1.76
	0 to 84	111	59.39	1.87	1.55 to 2.24
**HER2-positive**					
	<50	11	4.17	2.64	1.08 to 4.20
	50 to 84	25	13.70	1.83	1.08 to 2.57
	0 to 84	36	17.86	2.02	1.34 to 2.69
**HER2-negative**					
	<50	65	28.50	2.28	1.73 to 2.86
	50 to 84	154	100.77	1.53	1.28 to 1.77
	0 to 84	219	129.27	1.69	1.46 to 1.92
**ER+PR+**					
	<50	55	27.20	2.02	1.49 to 2.56
	50 to 84	173	95.89	1.80	1.52 to 2.08
	0 to 84	228	123.10	1.85	1.60 to 2.10
**ER-PR+**					
	<50	5	1.91	2.62	0.33 to 4.91
	50 to 84	12	5.74	2.09	0.84 to 3.35
	0 to 84	17	7.64	2.22	1.16 to 3.32
**ER+PR-**					
	<50	15	4.13	3.63	1.85 to 5.41
	50 to 84	24	16.31	1.47	0.88 to 2.06
	0 to 84	39	20.44	1.91	1.31 to 2.51
**ER or PR+ (luminal)**					
	<50	140	68.04	2.06	1.71 to 2.40
	50 to 84	437	247.27	1.77	1.61 to 1.94
	0 to 84	577	315.30	1.83	1.68 to 1.98
**ER or PR+ HER2+ (luminal HER2+)**					
	<50	6	2.68	2.24	0.48 to 4.00
	50 to 84	16	8.35	1.92	0.94 to 2.90
	0 to 84	22	11.03	2.00	1.15 to 2.84
**ER or PR+ HER2- (luminal HER2-)**					
	<50	53	23.49	2.26	1.65 to 2.87
	50 to 84	136	85.67	1.59	1.32 to 1.86
	0 to 84	189	109.15	1.73	1.48 to 2.00
**ER-PR- (non-luminal)**					
	<50	31	9.17	3.38	2.05 to 4.72
	50 to 84	41	29.10	1.41	0.97 to 1.85
	0 to 84	72	38.26	1.88	1.41 to 2.35
**ER-PR-HER2+ (non-luminal HER2+)**					
	<50	4	1.25	3.20	1.20 to 8.85
	50 to 84	6	4.52	1.33	0.35 to 2.29
	0 to 84	10	5.77	1.73	0.61 to 2.86
**ER-PR-HER2- (TN)**					
	<50	9	4.12	2.18	0.77 to 3.59
	50 to 84	15	12.55	1.20	0.60 to 1.79
	0 to 84	24	16.67	1.44	0.85 to 2.03

CI: confidence interval; ER: estrogen receptor; EXP: expected; FRR: familial relative risk; HER2: human epidermal growth factor receptor 2; PR: progesterone receptor; OBS: observed; TN: triple negative.

* <50: relatives were followed up from birth up until censoring or age 50, whichever occurred first, 50-84: from age 50 to censoring, 0-85: from birth to censoring

### Contribution of BRCA1 and BRCA2 mutations to FRR

Of the prevalent breast cancer patients screened for *BRCA1 *and *BRCA2 *mutations 519 had tumour pathology information. Among these, one carried a *BRCA1 *mutation, while six carried *BRCA2 *mutations. When these were excluded from the analyses, the estimated FRR for breast cancer by ER and PR status followed similar patterns to the overall analysis. For example, in analyses restricted to relatives followed up to 50 years of age, the FRR for ER-negative disease before and after excluding *BRCA1 *mutation carriers was unchanged (FRR = 3.5 vs 3.5 for ER-negative and FRR = 2.4 vs 2.4 for ER-positive disease). However, the number of breast cancer patients with mutation screening and pathology information was too small to estimate directly the precise contribution of *BRCA1 *and *BRCA2 *mutations to the FRRs by tumour subtype.

Instead, we estimated the contribution of *BRCA1 *and *BRCA2 *mutations to the breast cancer FRR by tumour subtype by modeling their effects in the risk prediction algorithm BOADICEA. The contribution of mutations in *BRCA1 *to the breast cancer FRR for relatives of ER-negative disease and ER-positive disease was estimated to be 24% and 1% respectively. The contribution of *BRCA2 *mutation to the breast cancer FRR was estimated to be 8.4% for relatives of both ER-positive and ER-negative patients. The contribution of mutations in *BRCA1 *to subtype specific FRR for ER-negative disease and ER-positive disease was 46% and 0.1% respectively.

### Contribution of recently identified susceptibility loci to FRR

The FRR for ER-positive and ER-negative disease due to each of the 12 recently identified breast cancer susceptibility loci and their estimated contribution to the FRR for breast cancer by tumour subtype are shown in Table 5. As expected, most of these SNPs result in larger FRRs for ER-positive than ER-negative disease. The SNPs were estimated to account for 1.9% of the breast cancer FRR in relatives of patients with ER-negative disease and for 9.6% of the breast cancer FRR in relatives of patients with ER-positive disease.

**Table 5:** Estimated FRR for ER-positive and ER-negative disease for recently identified breast cancer susceptibility loci

Locus	Ref	Genes in/near region	Variant	MAF	ER-negative				ER-positive			
					
					Hom- GRR	Het- GRR	FRR*	**%FRR**^**†**^	Hom- GRR	Het- GRR	FRR*	%FRR‡
10q26	[8]	*FGFR2*	rs2981582	0.38	1.18	1.08	1.0016	0.28	1.74	1.28	1.0186	3.08
16q12	[8]	*TNRC9/TOX3*	rs3803662	0.25	1.28	1.16	1.0036	0.63	1.48	1.25	1.0089	1.48
5q11	[8]	*MAP3KI*	rs889312	0.28	1.20	1.03	1.0009	0.16	1.26	1.12	1.0028	0.46
8q24	[8]	*FAM84B/c-MYC*	rs13281615	0.40	1.09	0.99	1.0003	0.05	1.29	1.11	1.0038	0.63
11p15	[8]	*LSP1*	rs3817198	0.30	1.13	1.01	1.0004	0.07	1.19	1.04	1.0010	0.17
3p24	[6]	*NEK10/SLC4A7*	rs4973768	0.46	1.12	1.06	1.0008	0.15	1.25	1.12	1.0031	0.52
17q22	[6]	*COX11*	rs6504950	0.27	1.06	1.03	1.0002	0.03	0.88	0.94	1.0008	0.13
10p14	[32]	*CASP8 (D302H)*	rs1045485	0.13	0.82	0.95	1.0004	0.07	0.83	0.89	1.0013	0.21
2q35	[62]	*TNP1/IGFBP5/IGFBP2/TNS1*	rs13387042	0.52	1.18	1.05	1.0018	0.31	1.29	1.08	1.0043	0.72
1p11.2	[11]	*NOTCH2/FCGR1B*	rs1124933	0.40	1.07	1.03	1.0003	0.05	1.42	1.22	1.0078	1.30
14q24.1	[11]	*RAD51L1*	rs999737	0.24	0.76	1.01	1.0005	0.09	0.69	0.93	1.0024	0.40
5p12	[11]	*MRPS30/FGFR10*	rs10941679	0.26	1.01	1.03	1.0001	0.01	1.18	1.17	1.0027	0.46

*Total*								1.90				9.56

ER: estrogen receptor; FRR: familial relative risk; GRR: genotypic relative risk; Het-GRR: GRR for heterozygotes (reference group: common homozygotes); Hom-GRR: GRR for rare homozygotes; MAF: minor allele frequency for populations of European ancestry; Ref: reference for MAF and GRR data.

* FRR due to susceptibility locus

† %FRR due to susceptibility locus- denominator is FRR for breast cancer for relatives of cases with ER-negative disease (1.78)

‡ %FRR due to susceptibility locus- denominator is FRR for breast cancer for relatives of cases with ER-positive disease (1.82)

## Discussion

We estimated breast cancer FRRs for relatives of breast cancer patients with different tumour subtypes using a cohort design and data from a population based study of breast cancer cases in the UK. The most important classification of breast cancers is that based on ER status of the tumour. Approximately 20% of the tumours in our cases were ER-negative and this proportion was similar whether or not a family member was also affected by breast cancer. Overall, we found no statistically significant difference in the FRRs for relatives of ER-negative patients and those for relatives of ER-positive cases. There was a suggestion that the FRR was higher for relatives of ER-negative cases for ages of the relative less than 50 years old, but the difference was not statistically significant (*P *= 0.07). We also observed a somewhat higher FRR for ER-positive disease when the relative was over the age of 50. Analysis of other tumour markers was consistent with the notion that FRR at older ages is driven by receptor positive tumours. There was no substantial familial risk of breast cancer for older relatives of TN cases.

Our results are consistent with published data, where for the most part no significant difference between FRR for breast cancer by ER status, or by joint ER/PR subtypes has been reported [33-42]. Some studies, for example Cotterchio et al. [34], reported a trend towards higher FRR for breast cancer in relatives of cases with ER PR-negative disease when the index case was younger. Other, smaller studies have reported a non-significantly higher FRR for relatives of cases with ER-positive disease, in particular when analyses were restricted to post-menopausal women [35,38,39]. Yang et al reported an association between family history and basal tumours [43]. We did not have data on basal markers in this study, but observed little increased FRR associated with triple negative disease. Our analysis differs from previous studies in that we were able to analyse the risk to relatives of breast cancer cases using a cohort approach. We found that the FRRs decreased by age of the relative, particularly for ER-negative disease.

Epidemiological studies of family history may be biased as patients with a family history of cancer may be more likely to take part or to recall their family history. Reporting of breast cancer in first degree relatives is generally considered to be accurate [44], however it is a shortcoming of this study that reported cancers in relatives could not be independently confirmed. It is unlikely that our results are influenced by a differential reporting bias by index cases harbouring tumours of different pathological subtype. A more important problem may be the accuracy of pathological data. In our data-set there is about 10% discordance between ER status derived from TMA analysis and medical records. However, separate analyses restricted to TMA data only or medical records data only yielded similar results (results not shown). Methodology and sensitivity of immunohistochemical methods, and cut-off points for ER positivity differ between different laboratories [45]. Some degree of non-differential misclassification is inevitable and may have obscured differences in FRR of different pathological subtypes. Misclassification may also result because sub-groupings are approximate and the most biologically relevant subtypes may have not been delineated.

Environmental risk factors for breast cancer vary by hormone-receptor status of the tumour. For example, nulliparity, late age at first birth, BMI, obesity among postmenopausal women, and early menarche have been more strongly linked to ER and/or PR-positive than ER-negative tumours [46,33,34,36,39,49]. These factors have been reported not to influence FRR for breast cancer [1] and were not included in our analyses. Interestingly, in our study the risk to sisters was slightly higher than that to mothers and this difference appeared to be restricted to relatives of breast cancer patients with ER-positive disease. It was previously suggested that the difference in risk between sisters and mothers reported in some studies may be due to a lower rate of breast cancer in mothers, who are by definition, parous [50]. However, temporal effects such as increased detection due to screening may also be relevant.

We estimated the effect of genetic variants on FRR for ER-negative and ER-positive breast cancer. The higher breast cancer FRR for younger relatives of ER-negative and PR-negative disease may be due to enrichment in *BRCA1 *as tumours from *BRCA1 *mutation carrier status very often arise in younger individuals and are ER and PR negative. This effect was still observed when analysis was restricted to a subset of non mutation carriers. However, the subset of breast cancer patients with both mutation screening and tumour pathology information was small and larger studies will be necessary to address this directly. In addition, the mutation screening methods used were not 100% sensitive [51] and some *BRCA1 *mutation carriers have been included in the analysis. Using a model based approach we estimated that about 24% of the breast cancer FRR to relatives of cases with ER-negative disease is due to *BRCA1 *mutations. *BRCA1 *and *BRCA2 *mutations together were estimated to explain 32% of breast cancer FRR for ER-negative disease and 9.4% of FRR for ER-positive disease. One limitation of these analyses is that the tumour type is known only for the index case and not the relative. It is therefore not possible to estimate the true subtype-specific FRRs (that is, the FRR that would occur if only that subtype occurred, in all relatives), or the contribution of each gene to these FRRs, since the concordance in pathology between relatives is not known. Assuming, as in our model, concordance in pathology between relatives is due solely to genotype, *BRCA1 *mutations explain about 46% of FRR for ER-negative disease.

Our estimates indicate that the 12 recently identified common breast cancer susceptibility alleles account for a larger proportion of the breast cancer FRR in relatives of patients with ER-positive than ER-negative disease. SNPs associated with ER-negative disease were estimated to contribute only 1.9% to the overall breast cancer FRR for relatives of patients with ER-negative disease, while SNPs associated with ER-positive disease were estimated to account for 9.6% of the breast cancer FRR for relatives of patients with ER-positive disease. Again, these estimates are approximate since they are based on the FRR for ER-positive and ER-negative index cases, but the pathology of the relatives is unknown (in effect, we assume concordance of the disease subtype in relatives). Results from studies of bilateral cancer suggest concordance in ER status between tumours arising against the same genetic background [52,53]. Future studies to estimate the FRRs using pathological subtypes in relatives would be worthwhile. Our calculations also assumed that these genetic variants interact multiplicatively on the risk of developing the disease. Although this seems a plausible assumption [54,55], it remains to be tested explicitly for the recently identified genetic variants. If the true model for the combined effects was additive, then the contributions to the subtype specific FRRs would be somewhat lower, 1.4% and 7.0% for ER-negative and ER-positive disease respectively.

Breast cancer risk is also influenced by rarer variants that confer moderate breast cancer risks such as *ATM *[56], *CHEK2 *[57], *BRIP1 *[58] and *PALB2 *[59]. There have been suggestions in a small number of studies that these variants may have stronger associations with ER-positive [60] or ER-negative [61] disease. It is estimated that this group of genes together contribute to approximately 2.3% of the overall familial relative risk.

The residual FRR for breast cancer may be driven by yet unidentified common variants that have similar patterns to those that have already been identified, that is more strongly associated with ER-positive than ER-negative disease, while GWAS restricted to ER-negative or *BRCA1 *mutation carriers may identify susceptibility variants associated with ER-negative disease. It is also possible that rare susceptibility variants with effects independent of or specific to tumour subtypes will be identified.

## Conclusions

We may conclude from our results that the FRR for breast cancer is significantly increased for each pathological subtype except TN tumours, although the numbers in the latter category were too small to draw definitive conclusions. When analyzed by tumour subtype, a surprisingly high proportion of FRR for ER-negative disease is already explained. We estimate that 32% of breast cancer FRR for ER-negative disease is explained by *BRCA1 *and *BRCA2 *mutations alone. Patients carrying such mutations may be advised to undergo prophylactic therapies such as oophorectomy or mastectomy. About 10% of the FRR for ER-positive disease is explained by 12 newly discovered SNPs, and the contributions of these SNPs to FRR are likely to be somewhat higher once the true causal variants are identified. The construction of informative risk prediction models for ER-positive disease is particularly important as the risk of ER-positive disease can be reduced by chemoprevention such as tamoxifen. It is possible that including novel genetic variants associated with breast cancer susceptibility in models may improve risk prediction for subtype specific disease.

## Abbreviations

BC: breast cancer; CI: confidence intervals; ECRIC: Eastern Cancer Registration and Information Centre; ER: estrogen receptor; FRR: familial relative risk; GRR: genotypic relative risk; GWAS: genome-wide association studies; HER2: human epidermal growth factor receptor 2; MAF: minor allele frequency; OR: odds ratio; PR: progesterone receptor; SEARCH: Studies of Epidemiology and Risk factors in Cancer Heredity; SNP: single nucleotide polymorphism; TN: triple negative; BOADICEA: Breast and Ovarian Analysis of Disease Incidence and Carrier Estimation Algorithm.

## Competing interests

The authors declare that they have no competing interests.

## Authors' contributions

NM, PDP, DFE and ACA contributed to the conception and execution of the project. NM carried out the analysis and manuscript writing. ACA and DFE supervised the analysis and participated in manuscript writing. Data were collated by Mitul Shah and the SEARCH team. DT and RM assisted in statistical analysis. FD, KD and EP generated immunohistochemistry data. All authors have read and approved the manuscript.

## Supplementary Material

Additional file 1 **Supplementary tables S1 and S2**. Table S1: Details of antibodies and scoring used for staining of tissue micro-arrays; Table S2: TMA and Data from the Eastern Cancer Registry and Information Centre (ECRIR) used for determination of ER status. Click here for file
