# Supplementary material for: Familial relative risks for breast cancer by pathological subtype: a population-based cohort study
Source: Breast Cancer Res. 2010 Feb 10;12(1):R10. doi: 10.1186/bcr2476 (PMC2880431; doi:10.1186/bcr2476)
Supplement: Additional file 1 — Supplementary tables S1 and S2. Table S1: Details of antibodies and scoring used for staining of tissue micro-arrays; Table S2: TMA and Data from the Eastern Cancer Registry and Information Centre (ECRIR) used for determination of ER status. [file bcr2476-S1.doc]

**Table S1. Details of antibodies and scoring used for staining of tissue micro-arrays**

|  | Supplier | Antibody | Dilution | Definition of positive |
| --- | --- | --- | --- | --- |
| ER | Novocastra | 6F11/2 | 1:70 | Sum of intensity (0-3) and percentage (0-5) >=3 |
| PR | Dako | PgR 636 | 1:50 | Sum of intensity (0-3) and percentage (0-5) >=3 |
| HER2 | Dako | Herceptest kit K5207 | pre-diluted | Intensity 2 or 3 |

**Table S2. TMA and Data from the Eastern Cancer Registry and Information Centre (ECRIR) used for determination of ER status**

|  | ECRIR data (medical records) | | |  |
| --- | --- | --- | --- | --- |
| TMA | ER-negative | ER-positive | unavailable | Total |
| ER-negative | 135 | 67 | 241 | 443 |
| ER-positive | 21 | 646 | 927 | 1,594 |
| Not done | 427 | 2065 | 0 | 2492 |
| Total | 583 | 2778 | 1168 | 4529 |
